# Assessment of Surgeon Judgment during Resection of Laryngeal Carcinoma

**DOI:** 10.22038/ijorl.2019.38025.2248

**Published:** 2020-07

**Authors:** Ebrahim Karimi, Reza Erfanian, Payman Dabirmoghadam, Saeed Shakiba, Saeed Sohrabpour

**Affiliations:** 1 *Otorhinolaryngology Research Center, Tehran University of Medical Sciences, Tehran, Iran.*; 2 *School of Medicine, Tehran University of Medical Sciences, Tehran, Iran. *

**Keywords:** Larynx, Squamous Cell Carcinoma, Microsurgery, Surgical margins

## Abstract

**Introduction:**

Carbon dioxide (CO2) laser surgery as a conservative tool plays a peculiar role in the management of head and neck cancer. Numerous patients who were candidates for transoral laryngeal microsurgery have forced us to eliminate frozen-section evaluation of surgical margins and use a magnified view of the larynx. The present study evaluated surgeon-judged negative margins with permanent microscopic pathologic evaluation.

**Materials and Methods:**

In this cross-sectional study, we evaluated the permanent pathologic margins of the resected laryngeal specimen which were considered negative by judgment of surgeons. Patients consisted of 61 pathologic proven T1-T2 laryngeal squamous cell carcinoma (SCC) cases. In all patients, tumor resection was performed via a transoral route with CO2 laser, and no residual laryngeal tumor was observed according to judgment of the surgeon. The patients with positive margin (s) underwent another resection. Patients were followed up for 18 months for tumor recurrence.

**Results:**

The obtained results demonstrated that pathologic margins were reported in 6 patients, with the deep margin being the most common positive margin. During the 18-month follow-up, 8 cases of recurrence were detected.

**Conclusion:**

Judgment of the surgeon was in agreement with permanent pathologic evaluation in transoral laryngeal laser resection at the early stages of laryngeal SCC in most cases. Nevertheless, it is suggested that further direct studies be conducted to evaluate the frozen section on oncologic outcomes in transoral laser surgery for laryngeal cancer.

## Introduction

Head and neck squamous cell carcinoma (HNSCC) is the eights leading cancer by incidence worldwide (1). Laryngeal squamous cell carcinoma accounts for about 1% of head and neck cancers. The larynx is divided into three anatomic regions: supraglottic, glottis, and subglottic. In each of these subunits, squamous cell carcinoma tumor can manifest different symptoms, such as dysphonia, dysphagia, or respiratory distress in obstructing lesions (2).

The timely treatment and complete eradication of this cancer with surgery plays a key role in the long-term prognosis of patients. There have been numerous studies investigating the efficacy and outcomes of various treatment modalities for early glottic malignancies (2-4). Laser surgery has been gaining increasing attention for the treatment of malignant tumors in the upper aerodigestive tract since the introduction of the carbon dioxide (CO2) laser into the field of current otolaryngologic applications (5).

Based on extensive studies, it is nowadays an acceptable practice to remove laryngeal lesions in the T1 and T2 stages by microscopic transoral surgery using laser as a regular method (2). The term early laryngeal cancer refers to carcinoma in situ, T1, and T2 of the larynx without positive cervical lymph node metastasis. Early-stage laryngeal neoplasms mostly arise from glottic or supraglottic regions, and the subglottic larynx is a less frequent primary tumor subsite (6,7). 

Microscopic methods have received increasing attention due to the complications and consequences of open surgeries, the need to spend more time and cost, as well as prolonged surgery duration (2,5). The complete removal of the lesion and resultant clear margins is recognized as the most critical delicate and important point in the resection of laryngeal tumors with laser CO2 (2,5). The presence of residual malignant cells in resection site due to incomplete removal with microscopic vision is found to be significantly associated with a high recurrence rate and consequently poor prognosis (2). On the other hand, the problems involved in the use of laser in the tumor removal, such as burning and carbonization of margins by laser-induced heat, would compromise the precise assessment of surgical resection margins (2,5). 

Therefore, numerous efforts have been made to find ways to ensure that the margin of the lesion is clean and free from tumor cells after transoral laryngeal microsurgery resection. Some of these methods include in vivo staining, immunofluorescence staining, narrow-band imaging based on the vascular bed and blood flow, and frozen section margin evaluation during operation to ensure complete removal of tumor

Moreover, a microscopic view during operation has seemingly a high correlation with the negative peripheral margin of the lesion (2,3,5,7-9).

The present study described the18-month follow-up of 61 patients with T1 and T2 laryngeal squamous cell carcinoma (SCC) who were treated with laser surgery, and margins were just evaluated by surgeon judgment during resection. 

## Materials and Methods

The present study was carried out on a total number of 61 patients who were pathologic proven cases of SCC of larynx in Amir Alam hospital in 2014. Their cancer stages were T1 or T2 according TNM staging system. The study was approved by the ethical committee (Approval number: XX.XXXX. REC. 1394. 1033) and an informed consent form was obtained. Before the commencement of the study, the patients were provided by the purpose of the study, and the informed consent was obtained; thereafter, they entered into the study. Demographic characteristics of the patients, including age, gender, and location of the primary lesion were recorded. Patients underwent surgical removal of laryngeal lesions with CO_2_ laser with high magnification. Neither did the patients have open surgery nor additional treatment, including radiotherapy. After the completion of tumor removal and aggressive rubbing of surgical site with brain cotton (for eliminating carbonated cells), cold-cup biopsy forceps was performed on five samples of margins (anterior, posterior, superior, inferior and depth of the surgical site), and they were sent for permanent pathologic evaluation. Frozen section evaluation of margin was not performed since one of the advantages of laser resection without reconstruction had been proposed as resection performed by a second-look procedure (2).

 The results of permanent pathological examinations were recorded and another resection with laser was carried out using second look procedure in case of positive margins on permanent pathologic evaluation. After discharge from hospital, the patients underwent regular follow-up laryngoscopy one month after the surgery and then every 3 months. It is worth noting that laryngeal biopsies were performed under general anesthesia at the suspicion of recurrence in the follow up examination.

## Results

Out of 61 patients enrolled in the present study, 57 cases were male and 4 subjects were female. The mean age of patients was reported as 56.9 years (30- 81 years). 44 patients had T1 tumors and 17 cases had T2 tumors. The location of the tumor was glottic in 42 patients, while the involved site was supraglottic in 19 patients. In 55 cases, all the samples sent from marginal laryngeal lesions were demonstrated to be free from cancer cells, and the resected margins were positive in 6(8.9%) subjects. For these patients, the positive marginal tumor was resected again the sample was sent to the pathology department for further evaluation. Deep margins were found to be the most common margin samples involved with cancer cells, followed by the samples of the anterior and inferior margins. During the 18-month follow-up, eight recurrences were detected (13.1% of cases), five of which were glottis cancer, and the other three were supraglottic cancer. The mean relapse time was obtained as 10 months, and the relapse was not associated with patients’ gender (P= 0.421) (chi-square test, square chi= 0.646). The patients who relapsed were candidates for radiotherapy and no recurrence was detected in further follow-up.Moreover, no significant relationship was observed between the primary site of the tumor and recurrence (P=0.6) (square chi test; 0.371 = square chi). Data analysis indicated no significant relationship between tumor T and its recurrence probability (P=0.431; chi square=0.2). On the other hand, there was no significant relationship between the stage and the recurrence time. Finally, there was no correlation between the status of histopathological margins and the relapse of the lesion (P=0.586, square chi=0.074).

## Discussion

The present study evaluated surgeon reliability to predict surgical margin status in transoral laser resection of T1 and T2 laryngeal cancer. The findings indicated that the difference in surgeons’ judgments about tumor margin using microscopic magnification was only 8.9%.

Today, the use of laser in the treatment of early stages of the laryngeal SCC is universally approved which is due to the success of this method in organ preservation and local tumor control. Nonetheless, it is of utmost importance to ensure that the lesion is completely removed from cancerous tissue with a safe margin (about 1-4 mm) (2).

To this end, various methods have been proposed, such as the frozen section procedure, use of in vivo staining, and an immunofluorescence staining, as well as the reliance on the surgeon's microscope vision using a high magnification of the microscope during surgery (2, 3, 7-9).

In a study conducted by Hendriksam et al., 15 patients with T1 and T 2 glottic SCC underwent surgical resection through transoral laser resection. They showed that the evaluation of the resected specimen margin had the worst correlation with the site of the tumor resection margins. They proposed that margin evaluation of resected specimen may be biased by thermal shrinkage of Laser beam and carbonization. Neither did they use the frozen section nor they mention clearly how they managed positive margins. The current study did not find the significantly worse outcome in positive margins and margin assessment technique was similar to techniques correlated with outcome. Three is a possibility that our approach will be different with positive margins (10).

In a study carried out by Wong et al. (2013), 29 patients (25 men, 4 women) underwent surgery based on surgeon's and were compared with the histopathological exams. The histopathologically examined margins were negative in 14 patients. In 10 patients, the margins of tumor were inconclusive due to burning and carbonization of the margin of the lesion. The results of histological studies demonstrated positive marginal involvement in 5 patients. It is worthy to note that five cases of recurrence were reported during the follow up period (9). Out of 61 cases that were included in the current study, only 6 patients had positive margins in the histopathological study. Moreover, there existed no carbonated inconclusive margins which may be attributed to sampling. In our technique, the resected specimen was not evaluated for histological examination, rather we analyzed the new specimens which were provided by cold cup forceps from margins of resected specimen after rubbing the site with brain cotton (for eliminating carbonized cells). Eight cases of recurrence were observed among 61 patients over a period of 18 months (13.6%). These cases received radiotherapy and no relapse was reported in the follow-up period. 

In a study carried out by Blanch et al. 2007, 357 patients who underwent transoral laryngeal microsurgery were assigned to three groups: negative margins, positive margins, and unknown margins; subsequently, relapsing was followed up for 24 months (11). There were 254 patients with a negative margin and a relapse rate of 22.4%. The relapse rate was obtained as 35.9% in cases with unknown margins, and recurrence occurred in 50% of cases with positive margins. Nevertheless, the recurrence was only 13.5% during the 18-month period in the current study.

Along the similar lines, a study was conducted by Fang et al. to evaluate the predictive value of in-operative frozen section in relapsing and survival of patients with SCC undergoing lesion removal using CO2 laser. The relapse was investigated in patients with a negative margins during the first transoral laryngeal microsurgery process and those requiring re-transoral laryngeal microsurgery due to positive frozen-section during surgery (8). 75 patients underwent surgical removal of SCC of larynx within 2004-2011, and the average follow-up was 33 months. 24 patients had a positive frozen section margin. The difference in the number of patients with positive margins in these studies is attributed to the false positivity of frozen section, in comparison with permanent pathology, and difference in the extent of surgery as we might resect more aggressive due to the absence of frozen section insurance. In those who had a negative margin for the first transoral laser microsurgery, organ preservation was 97% and a 5-year patient survival rate was reported as 84%. In One-year follow-up, 9 (12%) cases of relapse were reported in these patients (8). The positive margin in frozen section was found to be associated with recurrence. Therefore, it can be argued that the frozen section could not dramatically change the prognosis. In the present study, the percent of relapses was 13.5% during the 18-month period.

In a study performed by Piazza et al. on 147 patients with glottis in early-stage, the frozen section was not used during resection; however, the entire specimen was sent for permanent pathology evaluation. In the mentioned study, the patients had 26.5 positive margins; nonetheless, the important point was that they would follow a single positive superficial margin. It worth noting that similar to the present study, this study weakened the usage of the frozen section during surgery (12).

## Conclusion

As evidenced by the obtained results, laser can be widely used as a reliable method in the treatment of early stages of SCC in larynx. The achievement of proper local control and good prognosis with much less complications and much less time and cost is one of the advantages of this method over invasive surgical procedures or radiotherapy. The current study demonstrated than we can only rely on the surgeon judgment during the removal of the lesion by laser for the achievement of tumor-free margins.

In summary, microscopic magnified surgeon evaluation has good concordance with permanent pathologic evaluation in transoral laryngeal laser resection at early stages. Further direct studies need to evaluate the frozen section on oncologic outcomes in transoral laryngeal cancer resection. 
